# Relationship between Internet Use and Negative Affect

**DOI:** 10.1007/s11482-023-10158-z

**Published:** 2023-03-01

**Authors:** Hongyun Zheng, Wanglin Ma, Junpeng Li, Julio Botero

**Affiliations:** 1 College of Economics and Management, Huazhong Agricultural University, Wuhan, China; 2 Department of Global Value Chains and Trade, Faculty of Agribusiness and Commerce, Lincoln University, Christchurch, New Zealand; 3 School of Economics and Management, Huaiyin Normal University, Huai‘an, China; 4 Faculty of Agribusiness and Commerce, Lincoln University, Christchurch, New Zealand

**Keywords:** Internet use, Mental Health, Loneliness, Sadness, Life hardship, China, C26, I31, L86

## Abstract

While positive emotions like happiness and life satisfaction have received great attention, how to eliminate negative affect is largely neglected. This study contributes to the literature by examining the relationship between Internet use and people’s negative affect. Unlike previous studies that consider only one indicator, we capture negative affect from different dimensions by considering loneliness, sadness, and life hardship. We employ an endogenous ordered probit model to address the selection bias of Internet use and analyze the 20,107 individual-level samples sourced from the 2020 China Family Panel Studies survey. The results show that Internet use significantly reduces people’s loneliness, sadness, and life hardship. We also find that studying online and watching short videos would increase people’s loneliness feeling and shopping online deepens people’s life hardship. In contrast, using WeChat significantly reduces sadness and life hardship. Our findings confirm that guiding people to use the Internet appropriately is necessary to reduce negative affect and improve the quality of their life.

## Introduction

People always want to get up enthused about the new day’s prospects, and being a positive person is a simple desire for each of us. However, people’s subjective well-being is eroded by various unexpected life events, such as extreme weather events (Fluhrer & Kraehnert, 2022), epidemics (Rowan, 2022), and pressures from work and family life (Tauseef, 2021; Zheng et al., 2023). For example, the global epidemic of COVID-19 in 2019 and later on, the data reporting illness and deaths brought panic to people living in every corner of the world. It changed the ways of study, work, entertainment, and socialization. The World Happiness Report 2022 showed that laughing and smiling reduced significantly in most regions worldwide during the first two years (2020 and 2021) of the COVID-19 pandemic, while worry and sadness, two indicators of negative affect, increased dramatically during the first year (2020) of COVID-19 pandemic (Rowan, 2022).

There is a strong link between mental health and physical health, and the former leads to the latter, either directly or indirectly (Kesavayuth et al., 2021; Li et al., 2018; Ohrnberger et al., 2017). For example, Li et al. (2018) found that people with a higher level of happiness are more likely to have a higher body mass index (BMI) among urban Chinese adults. Kesavayuth et al. (2021) showed that a positive mental health condition could increase older people’s physical activities, reducing outpatient care in Australia. In contrast, negative affect (e.g., sadness, depression, and loneliness) are implicated in physical health problems. Ohrnberger et al. (2017) investigated the English population aged 50 years and older and found that people’s past mental health harms their present physical health. Because the negative affect leads to significant burdens for individuals, families, and the whole society (Doran & Kinchin, 2019), it is of essence to identify practical ways to help reduce people’s negative affects.

People’s actions to stressful life events (e.g., the pressures from work and family’s unexpected shocks) and the ways to address them are different (Colder Carras et al., 2018; Haslam et al., 2022). In the digital age, Internet use appears to play an increasingly important role in influencing people’s subjective well-being. It facilitates people’s information access through various channels and platforms, changes their working styles, and influences the ways people communicate with others (Castellacci & Tveito, 2018; Thomée, 2018). The COVID-19 pandemic has accelerated this trend. For example, social restrictions and isolation have increased online activities such as remote working and teaching (Akpınar, 2021; Llorente-Barroso et al., 2021; Wallinheimo & Evans, 2022). People are increasingly relying on the Internet for daily life. The last thing an increasing number of people do before going to sleep is to put down their phones to get off the Internet. Indeed, the importance of using the Internet in influencing mental conditions cannot be ignored.

This study aims to estimate the impact of Internet use on negative affect, utilizing the individual-level data sourced from the 2020 China Family Panel Studies (CFPS) survey. This is an interesting topic that has been overlooked. Although reducing negative affect is as important as enhancing positive emotions, most literature focuses on the latter (Bittmann, 2022; Jovanović & Joshanloo, 2022; Nie et al., 2021).

We attempt to make three contributions to the literature on mental well-being. First, we utilize loneliness, sadness, and life hardship to measure people’s negative affect from different dimensions. This is different from previous studies that focus on only one indicator, such as depression (Zhang et al., 2022), longingness (Wallinheimo & Evans, 2022), or weighted scores of mental health (Fan & Yang, 2022; Jung et al., 2022). Second, in addition to considering the Internet use status, we also explore how Internet use patterns influence people’s negative affect. Specifically, we consider five patterns: playing online games, studying online, using WeChat, shopping online, and watching short videos. Third, we utilize the endogenous ordered probit (EOP) model to address the selection bias issues. The EOP estimates the effect of a binary endogenous variable on an ordered outcome variable. It also corrects selection bias associated with observed and unobserved heterogeneities (Kawakatsu & Largey, 2009; Zhu et al., 2020).

A significant strand of literature has explored the relationship between Internet use and negative affect, such as mental depression, emotional problems, and loneliness (Ding et al., 2022; Fan & Yang, 2022; Golin, 2022; Hökby et al., 2016; Nowland et al., 2018; Silva et al., 2020; Thom et al., 2018; Wallinheimo & Evans, 2022; Yu et al., 2021; Zhang et al., 2022). However, the findings are still mixed. Hökby et al. (2016) found that the magnitude of Internet use among adolescents and young adults is negatively associated with mental health in some European countries, including Estonia, Hungary, Italy, Lithuania, Spain, and Sweden. Golin (2022) showed that broadband Internet leads to worse mental health for women (primarily those aged 17–30) but not for men in Germany. In contrast, some studies recorded a positive influence of Internet use on health outcomes. Ding et al. (2022) reported a positive association between mobile Internet use and a reduction in negative mental health during the COVID-19 pandemic in England. Wallinheimo and Evans (2022) showed that those who used the Internet more than once a day reported less loneliness feeling than those who used the Internet once a week or less.

A potential explanation for the mixed findings in the aforementioned studies might be the insufficiency in addressing the endogeneity of Internet use. People themselves decide to use the Internet. Their decisions are influenced by observed factors (e.g., age, educational experience, and social status) and unobserved factors (e.g., personal motivations and inabilities) (Fan & Salas Garcia, 2018; Ma & Wang, 2020). This fact leads to a potential endogeneity issue of Internet use. Previous studies have employed econometric approaches such as the Ordinary Least Square (OLS) model (Yuan, 2021) and between-groups analysis of covariance (ANCOVA) (Wallinheimo & Evans, 2022) for empirical analysis. However, those approaches cannot address the endogeneity issue of Internet use. Although Ding et al. (2022) employed the propensity score matching (PSM) model to address the selection bias, this approach only accounts for the observed selection bias. In compassion, estimating the impact of Internet use on people’s negative affect using the EOP model would add new insights.

The rest of this paper is organized as follows: Sect. 2 develops the research hypotheses and introduces the empirical model. Data sources, variable definitions, and descriptive statistics are reported in Sect. 3. The following Sect. 4 presents and discusses the empirical results. Finally, Sect. 5 concludes by discussing policy implications.

## Research Hypotheses and Empirical Model

### Research Hypotheses

The Internet attaches users to a vast web that can dilute stress, grief, and loneliness through multiple connections with others (Sims et al., 2017). With online social platforms, software, and applications (e.g., Instagram, Facebook, WeChat, and WhatsApp), people can efficiently communicate with others through e-mails, online chatting, and instant messages. Online communication channels save people from the awkwardness of the first meeting and thus encourage them to interact with strangers, making it possible for people to enrich their social networks. It is expected to reduce people’s isolation and promote their social integration. Accordingly, we propose the first hypothesis:

- *Hypothesis 1: Internet use reduces people’s loneliness.*

“*Shared sorrow is half a sorrow.*” It is widely agreed that communication is one of the few effective ways to release people’s psychic trauma (Elgar, 2013; Meier & Reinecke, 2021). Internet use is expected to possess the attribute of easing people’s sadness by promoting interpersonal communication (Ma & Wang, 2020; Ma & Zheng, 2022). For example, by using Instagram or WeChat, someone with a “broken” heart can find healing (i.e., pressure release) from online chatting with their fellows. Besides, the Internet provides online entertainment, interests, hobbies (e.g., movie-watching and online games), and psychological courses to people with bad moods, which helps people to release their negative emotions (Chopik, 2016). Based on the discussions here, we propose the second hypothesis as follows:

- *Hypothesis 2: Internet use reduces people’s sadness.*

Loneliness and sadness make people’s lives hard to go on. Then, if Hypotheses 1 and 2 are confirmed to be valid, Internet use can further mitigate people’s life hardships. Beyond this, Internet use can also reduce life hardship by improving people’s quality of life. Internet use strengthens people’s digital literacy and human capital (Lee et al., 2021; Paunov & Rollo, 2016) and increases their income generation and diversifies their consumption (Ma & Wang, 2020; Shahzad et al., 2020). Besides, Internet use improves people’s work and communication efficiency (Fan & Salas Garcia, 2018), making life more convenient and increasing leisure consumption. Therefore, we propose the third hypothesis:

- *Hypothesis 3: Internet use reduces life hardship.*

### Empirical Model

#### Modelling Internet use Decisions

This study assumes that a rational and risk-neutral respondent chooses to use the Internet to maximize the expected utility (Ankrah Twumasi et al., 2021; Ma & Zheng, 2022). Let ${U}_{1}$ proxy the utility of a respondent obtained from using the Internet and ${U}_{0}$ be the utility derived from not using. The respondents would choose to use the Internet if and only if they perceive a positive utility difference (${I}_{i}^{*}$) between using and non-using is greater than zero, i.e., ${I}_{i}^{*}={U}_{1}-{U}_{0}>0$. Although ${I}_{i}^{*}$ is unobservable, respondents’ decisions on Internet use can be modeled by the following latent variable model:

1 $$I_i^\ast=\gamma Z_i+\mu_i,with\;I_i=\left\{\begin{array}{cc}1,&if\;I_i^\ast>0\\0,&if\;I_i^\ast\leq0\end{array}\right.$$

where ${I}_{i}^{*}$ is a latent variable denoting the likelihood of using the Internet for respondent $i$. ${I}_{i}^{*}$ is determined by an observed dummy variable ${I}_{i}$. In particular, ${I}_{i}$ indicates the Internet use status (1 for Internet users and 0 for otherwise); ${Z}_{i}$ refers to a vector of control variables, such as age, gender, and working status, that are expected to affect respondents’ Internet use decisions; $\gamma$ refers to a vector of parameters to be estimated; and ${\mu }_{i}$ refers to the error term.

#### Modelling the Impact of Internet Use on Negative Affect

We then assume that loneliness, sadness, and life hardship, three indicators of negative affect, are linear functions of Internet use (${I}_{i}$) and other confounders (${X}_{i}$). The function can be modeled as follows:

2 $$\begin{array}{cc}{NA}_i^{S\ast}=\alpha I_i+\beta X_i+\varepsilon_i,&with\;{NA}_i^S=\left\{\begin{array}{cc}1&if\;{NA}_i^{S\ast}\leq C_1\\2&if\;C_1<{NA}_i^{S\ast}\leq C_2\\&\dots\\K&if\;C_{K-1}\leq{NA}_i^{S\ast}\end{array}\right.\end{array}$$

where ${NA}_{i}^{S*}$ is a latent variable indicating the level of the negative affect of respondent $i$, representing loneliness (*S* = 1), sadness (*S* = 2), and life hardship (*S* = 3). ${NA}_{i}^{S*}$ is unobserved and determined by an ordered categorical variable ${NA}_{i}^{S}$ and unknown cut-offs ${C}_{1}$, …, ${C}_{K-1}$, which together capture the level of a specific mental health outcome. Supposing $S\in\uppsi =\{1, 2, 3, 4\}$ and taking loneliness as an example here, ${NA}_{i}^{1}=1$ and ${NA}_{i}^{1}=4$ would indicate the lowest and highest levels of loneliness reported by individuals, respectively. ${I}_{i}$ represents Internet use status defined above; ${X}_{i}$ is a vector of exogenous variables; $\alpha$ and $\beta$ are parameters to be estimated; and ${\varepsilon }_{i}$ is the error term.

If the treatment variable, Internet use (${I}_{i}$), is randomly assigned, its impact on negative affect can be estimated using a simple ordered probit model specified by Eq. (2). However, Internet users and non-users may differ systematically, and these differences may induce observed and unobserved selection bias—a rigorous impact assessment cannot be obtained unless addressing these biases.

Previous studies have employed different econometric approaches to account for selection bias. In the scenario of analyzing the impact of an endogenous binary variable on discrete outcomes using cross-sectional data, scholars have employed the approaches such as the propensity score matching (PSM) method (Minah, 2022), the augmented inverse probability weighted (AIPW) estimator (Kurz, 2022), the inverse probability weighted regression adjustment (IPWRA) estimator (Grashuis & Skevas, 2022), and the endogenous ordered probit (EOP) model (Zheng & Ma, 2022). The PSM, AIPW, and IPWRA are powerful for mitigating the observed selection bias issue but not hidden selection bias. In comparison, the EOP model mitigates both observed and unobserved selection bias and estimates the binary treatment variable’s direct impact on the ordered outcomes (Kawakatsu & Largey, 2009; Zhu et al., 2020). Therefore, the EOP model is preferred in our study to evaluate the association between Internet use and negative affect outcomes.

### Endogenous Ordered Probit (EOP) Model

The EOP model estimations involve two stages: the first stage models people’s Internet use decisions (i.e., Eq. (1)), and the second stage models the impacts of Internet use and control variables on negative affect outcomes (i.e., Eq. (2)). The limited information maximum likelihood (LIML) estimator jointly estimates those two equations, and this procedure generates a correlation coefficient between the error terms in Eqs. (1) and (2), i.e., ${\rho }_{\mu \varepsilon }=\mathrm{corr}({\mu }_{i},{\varepsilon }_{i})$. A significant ${\rho }_{\mu \varepsilon }$ would indicate the presence of selection bias stemming from unobserved factors (Kawakatsu & Largey, 2009).

Following Kawakatsu and Largey (2009), consistent estimates of the EOP model can be obtained by implementing the following log-likelihood for the whole samples:

3 $$\mathrm{lnL}=\sum_{i=1}^{n}logPr({C}_{{F}_{i-1}}\le {MH}_{i}^{S*}<{C}_{{F}_{i}},\underset{\_}{{b}_{i}}\le {I}_{i}^{*}<\overline{{b}_{i}})$$

where $\left(\underset{\_}{{b}_{ij}},\overline{{b}_{ij}}\right)$ are $\mathrm{r}\times 1$ vectors, which contain ${j}^{th}$ element:

4 $$\left(\underset\_{b_{ij}},\overline{b_{ij}}\right)=\left\{\begin{array}{c}\left(-\infty,0\right)\;if\;I_{ij}=0\\\left(0,+\infty\right)\;if\;I_{ij}=1\end{array}\right.,j=1,\dots,r$$

For model identification, we need to introduce an identifying instrument variable (IV) into ${Z}_{i}$ but not ${X}_{i}$. In this study, a synthesized variable—the ratio of Internet users to the number of respondents (excluding the respondent) in the same county/district—is employed as the IV. The peer effect theory states that people’s decisions to adopt innovative technology, such as the Internet, tend to be inspired by the adoption status of their peers (e.g., neighbors, friends, relatives, and other villagers) (Tong & Zhu, 2020). Hence, people in a county/district with a high Internet penetration rate are more prone to Internet access. In addition, the synthesized IV would not directly influence people’s negative affect but through Internet use. Following previous studies (e.g., Adhvaryu & Nyshadham, 2017; Li et al., 2020), we conduct a falsification test to confirm the IV’s validity empirically. Specifically, we regress the IV and control variables on the three outcome variables and the treatment variable, respectively. The results of the falsification test (see Table 5 in the Appendix) suggest that the IV has a positive and significant correlation with Internet use. But it is uncorrelated with loneliness, sadness, and life hardship. The results verify the appropriateness of using the synthesized IV.

## Data, Variables, and Descriptive Statistics

### Data Source

This study utilizes the 2020 China Family Panel Studies (CFPS) data collected by the Institute of Social Science Survey (ISSS) at Peking University, Beijing, China. Using a multistage and random clustered design, the 2020 CFPS interviewed 28,590 respondents in 31 provinces of mainland China.^1^ The dataset comprises rich information on respondents’ socioeconomic, demographic, and household-level characteristics. The nationally representative and informative attributes make the CFPS a suitable dataset to analyze changes in China’s society (e.g., Green et al., 2021; Li & Zhou, 2020; Piketty et al., 2019; Zheng & Ma, 2021). For our study, the 2020 CFPS data provides detailed information on Chinese residents’ Internet usage and self-reported mental health outcomes, thereby supporting us in underscoring the impact of Internet use on negative affect. We drop observations with missing and extreme values during data cleaning. For example, we exclude respondents who did not report whether they have used the Internet. The final dataset for the empirical analysis comprises 20,107 observations.

### Variables

#### Negative Affect Outcomes

This study considers three negative affect outcomes: loneliness, sadness, and life hardship.^2^ In the survey questionnaire, the respondents were asked to answer three questions: “*How often do you feel lonely in the last week?*”; “*How often do you feel sad in the last week?*”; and “*How often do you feel that life is hard in the last week?*”. The answers to those questions were measured on a four-point Likert scale: 1 = Almost never (less than one day); 2 = Sometimes (1–2 days); 3 = Often (3–4 days); 4 = Most of the time (5–7 days). The respondents were asked to select one that best describes their negative emotions in the reference week.

#### Internet Use Status and Patterns

Internet use is the treatment variable. We consider both Internet use status and usage patterns in the present study. Specifically, the Internet use variable equals one if the respondent uses the Internet via smartphones and/or computers and zero otherwise. This definition is consistent with previous studies (Vatsa et al., 2022; Zheng et al., 2021). We consider five variables to capture respondents’ Internet use patterns: playing online games, studying online, using WeChat, shopping online, and watching short videos. They are all measured as binary variables, equaling one if Internet users reported taking the relevant activities and zero otherwise.

#### Control Variables

Drawing upon the existing studies on Internet use (Ma & Zhu, 2020; Vatsa et al., 2022; Zhang et al., 2019; Zheng et al., 2021) and negative affect (Akpınar, 2021; Chekroud et al., 2018; Lee et al., 2021; Sichel et al., 2022; Zhang et al., 2022), we selected a number of control variables to capture individual, household and contextual characteristics. They include respondents’ age, gender, education, working status, exercise, lunch break habit, reading experience, family size, elder ratio, child ratio, and living location. In particular, the respondents’ age, gender, education, working status, exercise, lunch break habit, and reading experience are included to reflect individual characteristics. For example, exercise participation is expected to reduce negative affect (Chekroud et al., 2018; Mikkelsen et al., 2017). Thus, we control respondents’ exercise participation to reflect this adverse relationship. Family size, elder ratio, and child ratio are included to capture households’ demographic and socioeconomic characteristics. Finally, substantial differences exist in terms of digital infrastructure, healthcare service access, employment opportunities, and economic conditions between urban and rural areas in China, which may lead to significant disparities in Internet use and negative affect. Therefore, we also include a dummy indicating respondents’ living locations.

### Descriptive Statistics

Table 1 reports the definitions and descriptive statistics of the variables used in this study. On average, the means of loneliness, sadness, and life hardship are 1.51, 1.55, and 1.25 out of 4, respectively, which are between the degrees of almost never (less than one day) and sometimes (1–2 days). The proportions of respondents with different negative affect outcomes are presented in Table 6 in the Appendix. It shows that the proportions of respondents who almost never experience loneliness, sadness, and life hardship are 62.86%, 55.74%, and 81.98%, respectively, representing the largest samples among all respondents. These statistics offer suggestive evidence to the findings of Xia and Yang (2019) and Li and Zhou (2020), who found that Chinese people tend to be beset by negative emotions. The proportion of Internet users in our sample is 63%, close to the national Internet penetration rate of 70% (NSBC, 2021). Regarding control variables, Table 1 shows that the respondents’ average age is about 47 years. Among them, 50% are male, 71% have worked in the reference week, 61% have the habit of lunch break, and 27% have read books in the reference year. On average, each household has around four members. The elder ratio and child ratio in surveyed households are 15% and 5%, respectively.

**Table 1:** Variable definitions and descriptive statistics

Variables	Definition	Mean	S.D
***Dependent variables***			
Loneliness	The frequency that a respondent feels lonely in the reference week: 1 = Almost never (less than one day); 2 = Sometimes (1–2 days); 3 = Often (3–4 days); 4 = Most of the time (5–7 days)	1.51	0.77
Sadness	The frequency that a respondent feels sad in the reference week: 1 = Almost never (less than one day); 2 = Sometimes (1–2 days); 3 = Often (3–4 days); 4 = Most of the time (5–7 days)	1.55	0.72
Life hardship	The frequency that a respondent feels that life is too hard to be continued: 1 = Almost never (less than one day); 2 = Sometimes (1–2 days); 3 = Often (3–4 days); 4 = Most of the time (5–7 days)	1.25	0.60
Internet use	1 if a respondent uses the Internet via smartphones and/or computers, 0 otherwise	0.63	0.48
***Independent variables***			
Age	Age of respondent (years)	47.28	15.45
Gender	1 if respondent is male, 0 otherwise	0.50	0.50
Education	Education experience of respondent^a^	3.02	1.47
Working status	1 if respondent worked in the reference week, 0 otherwise	0.71	0.45
Exercise	Frequency of participating in physical exercise in the reference year ^b^	2.55	2.34
Lunch break habit	1 if respondent has the habit of lunch break, 0 otherwise	0.61	0.49
Reading experience	1 if respondent has read at least one book in the reference year, 0 otherwise	0.27	0.44
Family size	Number of family members (persons)	4.22	2.06
Elder ratio	Ratio of the number of family members aged 65 or more years to family size	0.15	0.26
Child ratio	Ratio of the number of family members aged 0–14 years to family size	0.05	0.10
Urban	1 if respondent lives in the urban area, 0 otherwise	0.52	0.50
***Instrument variable***			
IV	Ratio of mobile Internet users to the number of respondents in the same county/district, excluding the respondent	0.66	0.14
Observations		20,107	

S.D. refers to the standard deviation

^a^ 1 = Illiterate; 2 = Primary school; 3 = Junior high school; 4 = Senior high school/secondary school/technical school/senior vocational school; 5 = 3-year college; 6 = 4-year college; 7 = Master’s program; 8 = Doctoral program

^b^ 1 = Never; 2 = Less than once per month; 3 = Less than once per week; 4 = 1–2 times per week; 5 = 3–4 times per week; 5 = 5–6 times per week; 7 = once per day; 8 = twice and more per day

Table 2 presents the mean values of selected variables categorized by Internet users and non-users, and the corresponding mean differences are reported in the last column. Generally, the results imply that the respondents are prone to systematically differ between Internet users and non-users. The mean differences in loneliness, sadness, and life hardship between Internet users and non-users are negative and statistically significant, indicating that Internet users are less likely to experience loneliness, sadness, and life hardship. Compared with non-users, Internet users tend to be younger, male, better educated, have a higher probability of working, do more physical exercise, read more books, and have fewer old family members. Table 2 strokes the preliminary relationship between Internet use and people’s negative affect. Notably, the simple mean comparison could not be regarded as solid as it does not control the effects of confounding factors. Given this, we then employ the EOP model to explore the impact of Internet use on negative affect.

**Table 2:** Mean differences in selected variables between Internet users and non-users

Variables	Internet users	Non-users	Mead difference
Loneliness	1.48 (0.72)	1.54 (0.85)	-0.06***
Sadness	1.53 (0.67)	1.57 (0.79)	-0.04***
Life hardship	1.19 (0.51)	1.34 (0.72)	-0.15***
Age	40.47 (12.91)	58.99 (12.06)	-18.53***
Gender	0.52 (0.50)	0.48 (0.50)	0.04***
Education	3.60 (1.38)	2.04 (1.03)	1.56***
Working status	0.76 (0.43)	0.62 (0.48)	0.14***
Exercise	2.74 (2.31)	2.23 (2.36)	0.51***
Lunch break habit	0.60 (0.49)	0.61 (0.49)	-0.01
Reading experience	0.38 (0.48)	0.09 (0.29)	0.28***
Family size	4.22 (2.00)	4.24 (2.15)	-0.02
Elder ratio	0.08 (0.18)	0.25 (0.33)	-0.16***
Child ratio	0.05 (0.10)	0.05 (0.10)	0.00
Urban	0.59 (0.49)	0.40 (0.49)	0.19***
IV	0.69 (0.15)	0.61 (0.12)	0.08***
Observations	12,714	7,393	

*** *p* < 0.01

The standard deviation is presented in parentheses

**Fig. 1:**
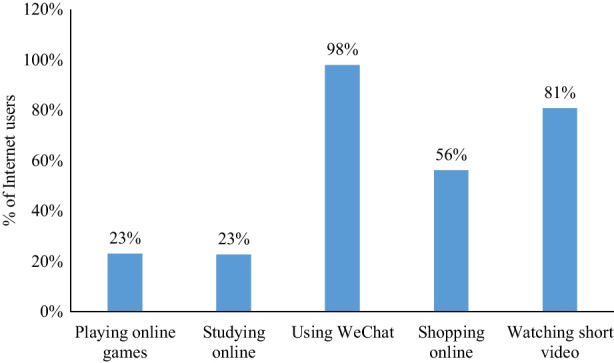
Distributions of Internet users with different utilization patterns

The distributions of Internet users (*N* = 12,714) with different utilization patterns are illustrated in Fig. 1. It shows that almost all Internet users (98%) have used WeChat, while 81% have watched short videos, indicating that instant communication and entertainments are most popular among Internet users. People who have studied and shopped online represent 23% and 56% of Internet users, respectively. Interestingly, the proportion of Internet users who have played online games only accounts for 23%. Thus, Internet users prefer communication and entertainment with no win or lose.

## Results and Discussion

Table 3 shows the estimation results of the EOP model. We first look at the estimated parameters, ${\rho }_{\mu \varepsilon }$, presented at the bottom of Table 3. It shows that ${\rho }_{\mu \varepsilon }$ is statistically significant in estimations for impacts on sadness, confirming the existence of selection bias associated with unobserved factors (Kawakatsu & Largey, 2009; Zhu et al., 2020). The significance of ${\rho }_{\mu \varepsilon }$ also justifies the necessity of employing the EOP model rather than the simple ordered probit model to address the selection bias issues. We discuss the first and second-stage estimations of the EOP model in the following two subsections.

**Table 3:** Impact of Internet use on the outcome variables: EOP estimates

	Impact on loneliness		Impact on sadness		Impact on life hardship	
	First-stage	Second-stage	First-stage	Second-stage	First-stage	Second-stage
Variables	Internet use (coefficients)	Loneliness (coefficients)	Internet use (coefficients)	Sadness (coefficients)	Internet use (coefficients)	Life hardship (coefficients)
Internet use		-0.113 (0.062)*		-0.194 (0.055)***		-0.268 (0.090)***
Age	-0.061 (0.001)***	-0.008 (0.001)***	-0.061 (0.001)***	-0.010 (0.001)***	-0.061 (0.001)***	-0.004 (0.002)**
Gender	0.067 (0.024)***	-0.047 (0.019)**	0.066 (0.025)***	-0.283 (0.019)***	0.067 (0.027)**	-0.191 (0.018)***
Education	0.339 (0.011)***	-0.060 (0.011)***	0.340 (0.013)***	-0.044 (0.007)***	0.339 (0.013)***	-0.109 (0.012)***
Working status	0.031 (0.028)	-0.033 (0.022)	0.032 (0.039)	0.002 (0.021)	0.031 (0.026)	-0.022 (0.025)
Exercise	0.056 (0.007)***	-0.011 (0.004)***	0.056 (0.005)***	-0.006 (0.004)	0.055 (0.006)***	-0.013 (0.005)***
Lunch break habit	0.141 (0.023)***	-0.017 (0.016)	0.141 (0.023)***	-0.026 (0.014)*	0.140 (0.027)***	-0.060 (0.020)***
Reading experience	0.355 (0.036)***	0.043 (0.018)**	0.353 (0.030)***	0.022 (0.020)	0.354 (0.037)***	-0.045 (0.032)
Family size	-0.026 (0.006)***	-0.052 (0.005)***	-0.026 (0.005)***	-0.023 (0.005)***	-0.026 (0.006)***	-0.017 (0.006)***
Elder ratio	0.099 (0.043)**	-0.020 (0.045)	0.100 (0.052)*	-0.035 (0.043)	0.098 (0.049)**	-0.180 (0.044)***
Child ratio	-0.140 (0.128)	-0.104 (0.090)	-0.135 (0.122)	-0.067 (0.083)	-0.138 (0.100)	-0.137 (0.111)
Urban	0.125 (0.025)***	-0.073 (0.021)***	0.126 (0.024)***	-0.041 (0.020)**	0.126 (0.030)***	-0.094 (0.020)***
IV	1.709 (0.120)***		1.707 (0.115)***		1.712 (0.121)***	
Constant	1.174 (0.247)***		1.176 (0.243)***		1.171 (0.329)***	
Provincial dummy	Yes	Yes	Yes	Yes	Yes	Yes
Cut points						
Cut 1		-0.556 (0.123)***		-0.755 (0.110)***		-0.016 (0.160)
Cut 2		0.440 (0.122)***		0.570 (0.109)***		0.809 (0.165)***
Cut 3		0.919 (0.124)***		1.055 (0.107)***		1.178 (0.165)***
${\rho }_{\mu \varepsilon }$	0.033 (0.032)		0.087 (0.031)***		0.066 (0.045)	
Observations	20,107		20,107		20,107	

The reference province is Beijing; 50-bootstrapped standard errors are presented in parentheses; *** *p* < 0.01, ** *p* < 0.05, and * *p* < 0.10

### Factors Affecting Internet Use

Columns 2, 4, and 6 of Table 3 present the results of the first-stage estimation of the EOP model, estimated by Eq. (1). The results help reflect the factors influencing people’s Internet use decisions. Because the first-stage estimations of the EOP model generate similar results, we focus on the results presented in column 2 in our interpretations for simplicity.

The age variable’s negative and statistically significant coefficient indicates that older respondents are less likely to use the Internet. Compared with their younger counterparts, older respondents lack the essential knowledge and skills to use new technologies, such as the Internet (Zhu et al., 2020). The gender variable has a positive and statistically significant coefficient, indicating that males are more likely to use the Internet than females. Other studies have reported similar findings (e.g., Sultana and Imtiaz, 2018; Su et al., 2020; Winker, 2005). The variable representing education, as expected, exerts a positive and significant impact on Internet use, suggesting that people with higher education levels are more likely to use the Internet. Educated people would learn Internet utilization skills easier and are more likely to perceive the benefits and services provided on the Internet, motivating them to use the Internet (Vatsa et al., 2022). We find a positive correlation between exercise and Internet use. Physical exercise improves human cognitive functions, stamina, and motivation (Tao et al., 2022), igniting people’s passion for trying new technologies.

Lunch break habit affects Internet use positively and significantly, indicating that people who rest during work are more likely to use the Internet. Those who know rest are likely to demand information on nurturing body and soul, motivating their adoption decisions. We observe positive and significant correlations between reading experience and Internet use. Given that the Internet allows people to access information, news, and opinion forums, it is understood that those with more reading experience are more likely to use the Internet than their counterparts. The negative coefficient of the family size variable suggests that respondents who live in a household with more members are less likely to use the Internet, which is in line with the findings of Zheng and Ma (2021). People in urban areas are more likely to use the Internet than rural respondents. This finding is expected as the number of Internet users in urban areas is twice more than in rural areas in China (Wang et al., 2021). Finally, a positive and significant correlation between IV and Internet use can be observed.

### Factors Affecting Negative Affect Outcomes

Columns 3, 5, and 7 of Table 3 report the results of the second stage estimations of the EOP model, that is, the factors affecting loneliness, sadness, and life hardship. We first discuss how Internet use influences negative affect before discussing the influences of control variables.

#### Impacts of Internet Use

The results reporting the impact of Internet use on people’s loneliness, sadness, and life hardship are presented in columns 3, 5, and 7 of Table 3, respectively. The negative and statistically significant coefficient of the Internet use variable on loneliness suggests that Internet users feel lonely lesser frequently than non-users, a finding that supports our Hypothesis 1. The negative association between Internet use and sadness suggests that Internet use can help to relieve people’s sad feelings. The finding echoes our Hypothesis 2. Finally, we obtain a negative and statistically significant coefficient of Internet use in column 7, demonstrating that Internet use contributes to alleviating people’s life hardships. The finding is consistent with Hypothesis 3, emphasizing that Internet use reduces life hardship.

#### Impacts of Control Variables

The results presented in Table 3 also provide valuable insights into the impact of control variables on negative affect. The age variable exerts a negative and statistically significant impact on loneliness, sadness, and life hardship, indicating that elders are less likely to experience negative affect than their younger counterparts. Older people are much better able to brush off life’s small stressors and be emotionally stable and compassionate. They can also make smart social decisions. Thus, older people have lower negative affect levels than their younger counterparts. Regarding gender, its negative impact on the three outcome variables suggests that men are less likely to experience negative sensations in their mental health. This echoes the findings of Zhang et al. (2022). Similarly, education is associated with a lower probability of experiencing loneliness, sadness, or life hardship. Education enables people to think positively and thus reduces the possibility of experiencing negative affect (Ding et al., 2022). As expected, exercise is another variable that has a significant and negative relationship with people’s loneliness and life hardship since exercise is essential in enhancing feelings of mental well-being (Ding et al., 2022).

Lunch break habit significantly reduces the level of sadness and life hardship, indicating that those who rest during work tend to be in positive mental conditions. Another interesting result is related to the reading experience, which indicates that the higher the probability of reading, the higher the likeliness of experiencing loneliness. Reading books enriches peoples’ emotions and gets people caught up in wild thoughts, which may lead to feelings of loneliness. As family size increases, people’s negative affect outcomes reduce. Family tie links family members together and improves the quality of life. Finally, people living in urban areas are less likely to experience loneliness, sadness, and life hardship.

### Associations Between Internet Use Patterns and Negative Affect Outcomes

While we find that Internet use reduces people’s negative affect, different Internet use patterns may exert a heterogenous effect. Given this, we further analyze the associations between Internet use patterns and negative affect outcomes among Internet users. For simplicity, we only report in Table 4 the results of the second-stage estimations of the EOP model.

**Table 4:** Impact of Internet use patterns on the outcome variables among Internet users: Second-stage estimation of the EOP model

Variables	Loneliness (coefficients)	Sadness (coefficients)	Life hardship (coefficients)
Playing online games	0.402 (0.294)	0.378 (0.376)	0.015 (0.243)
Control variables	Yes	Yes	Yes
Observations	12,714	12,714	12,714
Studying online	0.770 (0.195)***	0.775 (0.180)***	0.406 (0.288)
Control variables	Yes	Yes	Yes
Observations	12,714	12,714	12,714
Using WeChat	-0.235 (0.164)	-0.282 (0.145)*	-0.742 (0.259)***
Control variables	Yes	Yes	Yes
Observations	12,714	12,714	12,714
Shopping online	-0.099 (0.176)	-0.003 (0.156)	1.020 (0.167)***
Control variables	Yes	Yes	Yes
Observations	12,714	12,714	12,714
Watching short video	0.291 (0.163)*	0.122 (0.094)	0.229 (0.222)
Control variables	Yes	Yes	Yes
Observations	12,714	12,714	12,714

The reference province is Beijing; Standard errors are presented in parentheses; *** *p* < 0.01, ** *p* < 0.05, and * *p* < 0.10. For simplicity, the first-stage estimation results are not reported in Table 4

The results show that playing online games is positively associated with loneliness, sadness, and life hardship, but the effects are insignificant. Studying online is related to a greater feeling of loneliness and sadness. We can infer that the study involves social interaction and attending face-to-face lectures in schools and universities contribute to the development of networking and socialization (Akpınar, 2021; Ransom et al., 2022). Studying online removes the in-person component of the learning process, entailing a solo journey. WeChat is an essential tool to counteract the negative aspects of sadness and life hardship (Pang, 2018). Shopping online significantly deepens one’s life hardship. Shopping online allows one to access different goods and services offered worldwide, but some goods are beyond the purchasing power of Internet users. It may induce impulse purchases and empty Internet users’ wallets, potentially leading to a stronger sense of life hardship. Watching short videos is detrimental to loneliness. Let’s take into account how highly addictive these videos can be, the millions of users who access them on platforms like TikTok, and recent studies related to the effect of social media on adolescent depression. It is possible to determine that watching short videos is likely related to an increase in loneliness.

## Conclusions and Policy Implications

Improving people’s subjective well-being needs to consider both positive and negative sides. While positive emotions like happiness and life satisfaction have received great attention, how to eliminate negative affect outcomes is largely neglected. In this study, we focused on Internet use and explored its influence on people’s negative affect outcomes, captured by loneliness, sadness, and life hardship. We employed the endogenous ordered probit (EOP) model to address the selection bias of Internet use and estimate the individual data of 2020 China Family Panel Studies (CFPS). Further, we distinguished the specific Internet use patterns for potential heterogeneous insights.

The first-stage estimations of the EOP model show that people’s decisions to use the Internet are positively influenced by the individuals’ gender, educational experience, exercise and lunch break habits, reading experience, family elder ratio, and geographical locations. People’s age and family size are negatively associated with the probability of using the Internet. The second-stage estimation results reveal that Internet use significantly reduces respondents’ loneliness, sadness, and life hardship. Further analysis reveals that the effects of Internet use on negative affect vary across different patterns. Studying online increases loneliness and sadness, shopping online deepens life hardship, and watching short videos increases loneliness. In contrast, using WeChat significantly reduces sadness and life hardship.

Our results have practical implications for the Chinese government to achieve its goal of “meeting people’s longing for a better life”. First, our data shows that about 10% and 8% of people feel loneliness and sadness more than 3–4 days per week, indicating that negative affect has become an issue that cannot be ignored. Measures need to be taken to monitor changes in people’s negative affect and increase their senses of fulfillment, happiness, and security. We found that Internet use effectively mitigates negative affect outcomes, but the Internet adoption rate is around 63% based on the CFPS dataset. This means that around one-third still could benefit from Internet use when encountering negative mental issues. Thus, targeted policies and interventions are needed to encourage people to use the Internet. For example, as we found that elders and females are likely to be at a disadvantage in using the Internet, giving operation training on smartphones or computers to those people would be a practical way to increase the Internet use rate. In addition, those who live in rural rather than urban areas tend to have a lower probability of using the Internet. This indicates that more investment in Internet-based infrastructure construction is needed for rural areas.

In this study, we consider the general definition and specific usage pattern of Internet use. People may access the Internet through different channels, such as smartphones, tablets, and computers. Thus, future studies could explore whether accessing the Internet via different devices and their usage intensity has a heterogeneous impact on people’s subjective well-being. Self-reported subjective loneliness may be a temporary phantasm, while medical identification would provide a scientific judgment. Thus, understanding the relationship between Internet use and peoples’ mental health medically would help further generalize our understanding in this field.

## Data Availability

The data that support the findings of this study are available from Hongyun Zheng upon request. Not applicable

## Appendix 1

**Table 5:** Falsification test of the selected instrumental variable

Variables	${\chi }^{2}$-value	*p*-value
Loneliness	1.06	0.304
Sadness	2.69	0.101
Life hardship	1.60	0.206
Internet use	273.43***	0.000

^***^ *p* < 0.01

**Table 6:** Proportions of respondents with different negative affect outcomes

Variables	1 = Almost never (less than one day)	2 = Sometimes (1–2 days)	3 = Often (3–4 days)	4 = Most of the time (5–7 days)
Loneliness	62.86%	27.50%	5.80%	3.83%
Sadness	55.74%	36.67%	4.78%	2.80%
Life hardship	81.98%	13.52%	2.45%	2.05%

## Footnotes

1 The detailed sampling procedure can be found on the website http://www.isss.pku.edu.cn/cfps/en/index.htm.

2 Although indicators such as anxiety and fear can also reflect people’s negative affect, we did not consider them in this study due to the data unavailability.
